# Dementia and Driving

**DOI:** 10.1192/bjo.2024.528

**Published:** 2024-08-01

**Authors:** Mohammed Al-Dabbagh, Faquiha Muhammad, Dolapo Odegbaro

**Affiliations:** NHFT, Northampton, United Kingdom

## Abstract

**Aims:**

This audit focuses on assessing the compliance of health professionals with the UK law by informing the drivers with dementia about their legal requirement to report their condition to the DVLA and their insurance companies. The aim of this audit is to ensure public safety by adhering to the General Medical Council (GMC) guidance; “Confidentiality: patients’ fitness to drive and reporting concerns to the DVLA or DVA”, as well as the Driving with Dementia or Mild Cognitive Impairment Consensus Guidelines for Clinicians; endorsed by RCPsych and Alzheimer's Society. This will help ensure public safety and prevent potential accidents or incidents caused by impaired driving.

**Methods:**

The audit reviewed retrospective data of 40 patients selected randomly (17 males, 23 females and mean age 78 years old), referred to the memory clinic at Watermill Resource Centre in Berrywood Hospital, Northampton. The inclusion criteria was patients referred between 1st January and 31st December 2022 that were diagnosed with dementia. We set a compliance target of 100%.

**Results:**

The results showed that out of the 40 patients diagnosed with dementia, 23 had a recorded risk assessment. 11 patients were driving at the time of assessment. 7 patients were referred to occupational therapy for a driving assessment. The compliance in informing patients about reporting to the DVLA and their insurance companies was low. 8 out of 11 (73%) patients were informed about reporting to the DVLA, and 5 out of 11 (45%) were informed about contacting their insurance company. Additionally, only 4 out of 11 (36%) patients were informed about the consequences of not reporting to the DVLA and their insurer. There was also a lack of systematic documentation regarding driving risk assessment. There was no record of medics contacting the DVLA.

**Conclusion:**

Overall, the audit revealed a need for improvement in compliance and documentation. It is recommended that health professionals strictly adhere to their responsibilities in risk assessment and informing drivers with dementia about their legal requirements regarding informing DVLA and insurance companies. Clear documentation should be made using a standard template available.

